# Repurposing SGLT-2 Inhibitors as a Novel Therapeutic Strategy for Treatment-Resistant Meniere’s Disease

**DOI:** 10.3390/jpm15090412

**Published:** 2025-09-02

**Authors:** Sun-Uk Lee, Euyhyun Park

**Affiliations:** 1 Department of Neurology, Korea University College of Medicine, Seoul 02841, Republic of Korea; sulee716@gmail.com; 2 Neurotology Laboratory, Korea University Anam Hospital, Seoul 02841, Republic of Korea; 3 Department of Otorhinolaryngology-Head and Neck Surgery, Korea University College of Medicine, 73, Goryeodae-ro, Seongbuk-gu, Seoul 02841, Republic of Korea

**Keywords:** Meniere’s disease, SGLT-2 inhibitors, drug repurposing, endolymphatic hydrops, personalized medicine, treatment-resistant

## Abstract

**Background:** Meniere’s disease (MD) is a chronic inner ear disorder affecting approximately 0.2% of the population, with 30% of patients remaining refractory to conventional treatments. The pathophysiology involves endolymphatic hydrops, suggesting that agents affecting fluid homeostasis might provide therapeutic benefit. Sodium-glucose cotransporter 2 (SGLT-2) inhibitors, originally developed for diabetes, offer unique mechanisms including natriuresis and osmotic diuresis that may address the underlying fluid imbalance in MD. **Methods**: We conducted a retrospective observational study at the Korea University Anam Hospital, analyzing the medical records of patients with definite MD (Bárány Society criteria) who received off-label empagliflozin 10 mg daily between January 2023 and December 2023. Six patients (3 men, 3 women; mean age 55.8 years) with treatment-resistant MD were identified who had failed conventional therapy for at least 3 months. Primary outcomes included changes in pure tone threshold average (PTA), low-frequency threshold average (LFA), vertigo episode frequency, and vertigo severity using visual analog scale (VAS) scores, assessed at baseline and after 3 months of treatment. **Results:** All patients demonstrated clinically significant improvements in both auditory and vestibular symptoms. Mean PTA improved from 31.4 dB to 20.8 dB (improvement of 10.6 dB, *p* < 0.05). Low-frequency hearing showed more substantial recovery, with LFA improving from 37.2 dB to 15.6 dB (improvement of 21.6 dB, *p* < 0.01). Vertigo frequency decreased dramatically from 1.6 episodes per month to 0.1 episodes per month, with four patients experiencing a complete resolution of vertigo episodes. VAS scores for vertigo severity decreased from 5.2 to 0.5. Treatment was well-tolerated, with only minor adverse effects reported in two patients: transient polyuria in one patient and 5 kg weight loss in another, both consistent with the known pharmacological profile of SGLT-2 inhibitors. **Conclusions**: This preliminary study suggests a potential clinical benefit of repurposing SGLT-2 inhibitors for treatment-resistant MD. However, the retrospective design and inherent limitations prevent definitive conclusions about causality. The significant improvements observed in both hearing thresholds and vestibular symptoms warrant further investigation through randomized controlled trials with objective outcome measures to establish the true efficacy of this therapeutic approach.

## 1. Introduction

Meniere’s disease (MD) is a chronic inner ear disorder characterized by episodic vertigo, fluctuating sensorineural hearing loss, tinnitus, and aural fullness [1]. With a prevalence of approximately 0.2% globally [2], MD typically manifests in middle age and represents a significant cause of disability due to unpredictable vertigo attacks that severely impact quality of life and employment [3].

The pathophysiology of MD centers on endolymphatic hydrops, an excessive accumulation of endolymph within inner ear compartments, first histologically demonstrated by Hallpike and Cairns in 1938 [4]. Modern gadolinium-enhanced magnetic resonance imaging has enabled the in vivo visualization of endolymphatic hydrops, confirming its presence in most MD patients [5]. Recent research has revealed MD as a complex multifactorial disease involving genetic, immunological, and environmental factors, with distinct inflammatory signatures including elevated pro-inflammatory cytokines [6,7].

Current management follows a stepwise approach, beginning with lifestyle modifications and conservative treatments. First-line therapies include dietary sodium restriction, diuretics, and betahistine [8]. However, evidence for conventional treatments remains limited, with systematic reviews highlighting insufficient high-quality data [9]. The landmark BEMED trial failed to demonstrate the significant benefits of betahistine over placebo [10], and meta-analyses showed only modest evidence for diuretic efficacy [11]. Despite these approaches, approximately 30% of patients fail to respond adequately to conventional treatment, highlighting the urgent need for novel therapeutic strategies [12].

Sodium-glucose cotransporter 2 (SGLT-2) inhibitors represent a promising therapeutic candidate for MD through several mechanisms relevant to inner ear pathophysiology. These agents work by selectively inhibiting SGLT-2 receptors in the kidney, blocking glucose reabsorption and leading to glucosuria with natriuresis and osmotic diuresis [13]. Beyond glucose lowering, SGLT-2 inhibitors demonstrate pleiotropic effects including the following: (1) gradual and sustained volume reduction through physiological diuresis, potentially providing more stable inner ear fluid homeostasis compared to traditional diuretics; (2) anti-inflammatory properties, reducing circulating pro-inflammatory cytokines that may contribute to MD pathogenesis; and (3) microvascular improvements through enhanced endothelial function [14,15].

The drug repurposing approach offers significant advantages for rare conditions like refractory MD, with established safety profiles potentially accelerating clinical evaluation [16]. While a recent preclinical study found empagliflozin ineffective in preventing hydrops development in mice, their study focused on prevention rather than treatment of the established disease, and clinical translation remains to be determined [17].

To our knowledge, this represents the first clinical investigation exploring SGLT-2 inhibitor repurposing for established MD treatment in humans. This retrospective analysis examines clinical outcomes in patients who received off-label SGLT-2 inhibitor therapy after failing conventional treatment, providing preliminary evidence to inform future prospective trials.

## 2. Methods

### 2.1. Study Design and Participants

This retrospective study was conducted using clinical data from patients treated at the Neuroscience Center, under the approval of the Institutional Review Board of Korea University Anam Hospital (IRB No. 2023AN0297, approval date: 10 July 2023). The IRB approval was part of a broader institutional registry protocol. A continuing review approval was obtained on 20 June 2024. Medical records were reviewed for patients with definite MD according to the Bárány Society criteria who received off-label SGLT-2 inhibitor treatment between January 2023 and December 2023 [18].

Six patients (3 men, 3 women) with definite MD were identified who had documented failure to respond to conventional treatment, defined as persistent symptoms despite at least 3 months of standard treatment approaches, including diuretics and/or betahistine. These patients were subsequently prescribed off-label empagliflozin based on clinical judgment considering the theoretical mechanism of action and refractory nature of their condition. None of the patients had diabetes mellitus, metabolic syndrome, chronic kidney disease, or heart failure at the time of treatment initiation. Baseline clinical characteristics of the patients are presented in Table 1.

### 2.2. Treatment Protocol

Patients with definite MD who had documented failure to respond to conventional treatment were transitioned directly to empagliflozin 10 mg orally once daily without a formal washout period. This approach was chosen to prevent potential symptom exacerbation during treatment interruption in these refractory patients. Vestibular suppressants (dimenhydrinate 50 mg or meclizine 25 mg) were permitted as rescue medication for acute vertigo episodes as needed.

### 2.3. Data Collection and Outcome Measures

Medical records were systematically reviewed to extract data on the following:

Primary outcome measures:

1. Change in pure tone threshold average (PTA), calculated as the average of thresholds at 0.5, 1, 2, and 4 kHz using the formula: (0.5 kHz + 2 × 1 kHz + 2 × 2 kHz + 4 kHz)/6.
2. Change in low-frequency threshold average (LFA), calculated as the average of thresholds at 0.25, 0.5, and 1 kHz.
3. Change in the frequency of definitive vertigo episodes per month.
4. Change in vertigo severity using a visual analog scale (VAS) consisting of a 100 mm horizontal line with anchor points of 0 (‘no vertigo symptoms’) and 10 (‘worst possible vertigo symptoms’). Patients were instructed to mark their average symptom severity without numerical reference points visible.
5. Treatment-related adverse effects.

Assessments were performed at baseline and after 3 months of treatment based on available clinical records.

### 2.4. Statistical Analysis

Given the small sample size (n = 6), descriptive statistics were calculated for outcome measures. Pre- and post-treatment comparisons were performed using the Wilcoxon signed-rank test for paired non-parametric data, as normality could not be assumed with the small sample size. Statistical significance was set at *p* < 0.05. All statistical analyses were performed using SPSS version 28.0 (IBM Corp, Armonk, NY, USA). We acknowledge that the limited sample size substantially reduces statistical power and increases the risk of both type I and type II errors.

## 3. Results

### 3.1. Efficacy Outcomes

All patients demonstrated significant improvements in hearing thresholds and vertigo symptoms after 3 months of SGLT-2 inhibitor therapy. Mean PTA improved from 31.4 dB to 20.8 dB (improvement of 10.6 dB, *p* < 0.05). Low-frequency hearing showed more substantial recovery, with LFA improving from 37.2 dB to 15.6 dB (an improvement of 21.6 dB, *p* < 0.01). Vertigo frequency decreased dramatically from 1.6 episodes per month to 0.1 episodes per month, with four patients experiencing a complete resolution of vertigo episodes. VAS scores for vertigo severity decreased from 5.2 to 0.5. Detailed outcomes are presented in Table 2.

The improvement in LFA was particularly notable, with a mean reduction of 21.6 dB, which is clinically significant for patients with Meniere’s disease who typically experience greater hearing loss in the low frequencies. Four of the six patients experienced a complete resolution of vertigo episodes.

### 3.2. Safety Outcomes

Overall, SGLT-2 inhibitor therapy was well-tolerated. One patient experienced mild polyuria during the first week of treatment, which resolved spontaneously without intervention. Another patient experienced a 5 kg weight loss over the 3-month treatment period, which is consistent with the known effects of SGLT-2 inhibitors. No episodes of hypoglycemia, urinary tract infections, or genital infections were reported. All patients completed the full course of treatment with 100% adherence.

## 4. Discussion

This retrospective analysis suggests that repurposing SGLT-2 inhibitors may be effective in improving both auditory and vestibular symptoms in patients with MD who are refractory to conventional treatment. Patients showed robust improvement in vertigo frequency and hearing thresholds, with the complete resolution of vertigo episodes in four of six patients.

The beneficial effects of SGLT-2 inhibition may be attributed to several mechanisms. SGLT-2 inhibitors induce natriuresis and osmotic diuresis, which could reduce systemic fluid volume and, consequently, endolymphatic pressure [19]. Unlike traditional diuretics, SGLT-2 inhibitors promote more gradual and sustained volume reduction, potentially providing more stable fluid homeostasis [20]. This physiological approach to diuresis may be particularly advantageous in MD, where sudden changes in fluid balance could potentially exacerbate symptoms. Additionally, SGLT-2 inhibitors have demonstrated pleiotropic effects, including improvements in vascular function and reduction in oxidative stress, which may address microvascular hypoperfusion as a potential pathomechanism of MD [21].

Recent advances in understanding inner ear physiology have revealed the presence of glucose transporters in cochlear and vestibular tissues. The inner ear’s glucose metabolism is critical for maintaining cellular energy homeostasis, particularly in hair cells and supporting cells that are metabolically active [22]. SGLT-2 inhibitors may influence inner ear glucose handling and fluid balance through both direct effects on local glucose transporters and indirect effects via systemic metabolic changes. The reduction in systemic glucose levels and associated osmotic effects may contribute to decreased endolymphatic volume and pressure.

The anti-inflammatory properties of SGLT-2 inhibitors represent another potential therapeutic mechanism. Recent research has identified inflammatory processes as key contributors to MD pathogenesis, with elevated levels of pro-inflammatory cytokines found in MD patients [6,7]. SGLT-2 inhibitors have been shown to reduce circulating inflammatory markers, potentially addressing the inflammatory component of MD pathophysiology.

The improvement in the low-frequency hearing thresholds is particularly significant, as these frequencies are typically most affected in MD [23]. This finding suggests that SGLT-2 inhibition may address the underlying pathophysiology of endolymphatic hydrops rather than simply masking symptoms. The preferential improvement in low-frequency hearing aligns with the expected effects of reducing endolymphatic pressure, as low-frequency hearing is most sensitive to changes in inner ear fluid dynamics [24].

Traditional diuretics used in MD management primarily target the distal convoluted tubule and collecting duct, and can cause electrolyte imbalances with inconsistent effects on endolymphatic homeostasis [25]. In contrast, SGLT-2 inhibitors offer a more physiological approach to diuresis by blocking glucose reabsorption in the proximal tubule, leading to glucosuria-induced osmotic diuresis [26]. This mechanism may provide more consistent fluid balance effects compared to traditional diuretics, potentially explaining the dramatic reduction in vertigo episodes observed in our patients.

The concept of personalized medicine in MD management is gaining recognition, with acknowledgment that different patients may respond to different therapeutic approaches based on their underlying pathophysiology [27]. Patients with predominantly fluid retention-related symptoms may be ideal candidates for SGLT-2 inhibitor therapy, particularly those with comorbid metabolic conditions. Future research should focus on identifying biomarkers or clinical characteristics that can predict the response to SGLT-2 inhibitor therapy, enabling more targeted treatment approaches.

The drug repurposing approach offers several advantages in addressing unmet medical needs in rare conditions like refractory MD. SGLT-2 inhibitors have well-established safety profiles from extensive use in diabetes management, potentially accelerating their evaluation for alternative indications [28]. Moreover, the existing clinical experience provides valuable insights into safety profiles, drug interactions, and contraindications. The cost-effectiveness of repurposing existing medications compared to developing novel therapies represents another significant advantage, particularly important for rare conditions where pharmaceutical investment may be limited. The excellent tolerability profile observed in our study is consistent with the known safety characteristics of SGLT-2 inhibitors. The mild adverse effects, including transient polyuria and modest weight loss, are generally manageable and may even provide additional benefits for patients with comorbid conditions such as hypertension or obesity. This tolerability advantage over traditional treatments may improve patient adherence and long-term treatment outcomes.

This study has several important limitations that must be acknowledged. First, the retrospective observational design without a control group fundamentally limits our ability to establish causality between SGLT-2 inhibitor treatment and clinical improvements. The natural fluctuating course of MD makes it particularly challenging to distinguish treatment effects from natural disease progression or placebo effects. Second, the absence of objective measures such as gadolinium-enhanced MRI to assess endolymphatic hydrops represents a critical limitation [5,29]. Third, the small sample size (n = 6) and relatively short follow-up period (3 months) limit the generalizability of findings and prevent the assessment of long-term treatment sustainability. Fourth, the lack of a formal washout period means that potential residual effects from previous treatments cannot be completely excluded. Fifth, the discordance with preclinical findings by Pålbrink et al., who found empagliflozin ineffective in preventing endolymphatic hydrops in a mouse model, raises questions about mechanism and translational relevance, though their study focused on prevention rather than treatment of the established disease.

Future research should prioritize randomized controlled trials with adequate sample sizes and appropriate control groups to establish definitive efficacy and safety profiles. Studies should incorporate objective outcome measures, including gadolinium-enhanced MRI assessment of endolymphatic hydrops, vestibular function testing, and validated quality of life instruments. Long-term follow-up studies are essential to determine treatment durability and identify potential late adverse effects.

The investigation of optimal dosing regimens, treatment duration, and patient selection criteria for SGLT-2 inhibitor therapy in MD represents another critical research priority. Pharmacokinetic studies examining the penetration of these agents into inner ear fluids would provide valuable mechanistic insights. Additionally, combination therapy approaches utilizing SGLT-2 inhibitors with other treatments may offer synergistic benefits and should be explored.

The development of biomarkers to predict treatment response would significantly advance personalized medicine approaches in MD management. This could include genetic markers, inflammatory profiles, or metabolic parameters that identify the patients most likely to benefit from SGLT-2 inhibitor therapy.

Despite these limitations, our findings provide compelling preliminary evidence supporting the potential repurposing of SGLT-2 inhibitors as a therapeutic candidate in treatment-resistant MD, warranting further investigation through properly designed clinical trials.

## 5. Conclusions

This preliminary retrospective study suggests that SGLT-2 inhibitor therapy may have potential benefits for patients with treatment-resistant MD. However, the observational design and inherent limitations prevent definitive conclusions about causality. The significant improvements observed in both hearing thresholds and vestibular symptoms warrant further investigation through properly designed randomized controlled trials with objective outcome measures, including gadolinium-enhanced MRI assessment of endolymphatic hydrops, to establish the true efficacy of this therapeutic approach.

## Figures and Tables

**Table 1:** Baseline characteristics of patients with refractory Meniere’s disease.

Patient	Age/Sex	Disease Duration (Months)	Affected Ear	Previous Treatment	Duration of Previous Treatment (Months)
1	45/M	12	Right	HCTZ 25 mg QD + Amiloride 5 mg QD + Betahistine 24 mg BID	6
2	67/F	6	Right	HCTZ 25 mg QD + Amiloride 5 mg QD + Betahistine 24 mg BID	3
3	65/F	12	Right	HCTZ 25 mg QD + Betahistine 24 mg BID	7
4	39/F	14	Right	HCTZ 25 mg QD + Betahistine 24 mg BID	6
5	56/M	19	Left	HCTZ 25 mg QD + Amiloride 5 mg QD + Betahistine 24 mg BID	6
6	63/M	10	Left	Isosorbide mononitrate 10 mL BID	10

Abbreviations: HCTZ, hydrochlorothiazide; QD, once daily; BID, twice daily.

**Table 2:** Changes in hearing thresholds and vertigo parameters after 3 months of SGLT-2 inhibitor treatment.

Parameter	Baseline	After 3 Months	Mean Change
Pure-tone average, dB *	31.4 (15.0–45.0)	20.8 (4.2–40.8)	−10.6
Low-frequency average, dB †	37.2 (16.7–55.0)	15.6 (5.0–28.3)	−21.6
Vertigo episodes per month	1.6 (1.0–3.3)	0.1 (0–0.3)	−1.5
Vertigo severity (VAS score, 0–10)	5.2 (3–7)	0.5 (0–2)	−4.7

* Pure-tone average was calculated as the average of thresholds at 0.5, 1, 2, and 4 kHz using the formula: (0.5 kHz + 2 × 1 kHz + 2 × 2 kHz + 4 kHz)/6. † Low-frequency average was calculated as the average of thresholds at 0.25, 0.5, and 1 kHz. Data are presented as mean (range). Abbreviation: VAS, visual analog scale.

## Data Availability

The data that support the findings of this study are available from the corresponding author upon reasonable request. Due to privacy and ethical considerations, certain restrictions may apply to the availability of data.
